# *N*′-[(*E*)-1-(4-Bromo­phen­yl)ethyl­idene]-2-(2-methyl-4-nitro-1*H*-imidazol-1-yl)acetohydrazide

**DOI:** 10.1107/S160053681202795X

**Published:** 2012-06-23

**Authors:** Hoong-Kun Fun, Ching Kheng Quah, Priya V. Frank, N. Damodara, Balakrishna Kalluraya

**Affiliations:** a X-ray Crystallography Unit, School of Physics, Universiti Sains Malaysia, 11800 USM, Penang, Malaysia; b Department of Studies in Chemistry, Mangalore University, Mangalagangotri, Mangalore 574 199, India; c Department of Chemistry, Canara Engineering College, Mangalore 574 219, India

## Abstract

In the title compound, C_14_H_14_BrN_5_O_3_, the mean plane of the imidazole ring (r.m.s deviation = 0.004 Å) forms a dihedral angle of 58.13 (7)° with the benzene ring. In the crystal, mol­ecules are linked *via* N—H⋯O, C—H⋯O and C—H⋯N hydrogen bonds into a three-dimensional network. A short Br⋯Br contact of 3.4932 (2) Å also occurs.

## Related literature

For general background to and applications of imidazole derivatives, see: Priya & Kalluraya (2005 ▶); Krapcho & Turk (1966 ▶); Chu *et al.* (2004 ▶); Khalafi-Nezhad *et al.* (2005 ▶). For standard bond-length data, see: Allen *et al.* (1987 ▶). For the stability of the temperature controller used in the data collection, see Cosier & Glazer (1986 ▶).
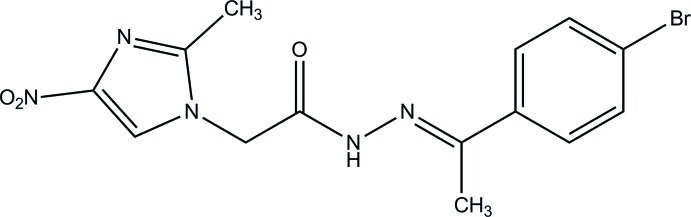


## Experimental

### 

#### Crystal data

C_14_H_14_BrN_5_O_3_
*M* *_r_* = 380.21
Monoclinic, 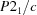

*a* = 8.4176 (1) Å
*b* = 10.6541 (1) Å
*c* = 17.4933 (2) Å
β = 90.100 (1)°
*V* = 1568.83 (3) Å^3^
*Z* = 4
Mo *K*α radiation
μ = 2.64 mm^−1^
*T* = 100 K
0.37 × 0.33 × 0.15 mm

#### Data collection

Bruker SMART APEXII CCD area-detector diffractometer
Absorption correction: multi-scan (*SADABS*; Bruker, 2009 ▶) *T*_min_ = 0.441, *T*_max_ = 0.701
45099 measured reflections
6320 independent reflections
5026 reflections with *I* > 2σ(*I*)
*R*_int_ = 0.040

#### Refinement

*R*[*F* ^2^ > 2σ(*F* ^2^)] = 0.032
*wR*(*F* ^2^) = 0.082
*S* = 1.02
6320 reflections
214 parameters
H atoms treated by a mixture of independent and constrained refinement
Δρ_max_ = 0.88 e Å^−3^
Δρ_min_ = −0.66 e Å^−3^

### 

Data collection: *APEX2* (Bruker, 2009 ▶); cell refinement: *SAINT* (Bruker, 2009 ▶); data reduction: *SAINT*; program(s) used to solve structure: *SHELXTL* (Sheldrick, 2008 ▶); program(s) used to refine structure: *SHELXTL*; molecular graphics: *SHELXTL*; software used to prepare material for publication: *SHELXTL* and *PLATON* (Spek, 2009 ▶).

## Supplementary Material

Crystal structure: contains datablock(s) global, I. DOI: 10.1107/S160053681202795X/rz2774sup1.cif

Structure factors: contains datablock(s) I. DOI: 10.1107/S160053681202795X/rz2774Isup2.hkl

Supplementary material file. DOI: 10.1107/S160053681202795X/rz2774Isup3.cml

Additional supplementary materials:  crystallographic information; 3D view; checkCIF report

## Figures and Tables

**Table 1:** Hydrogen-bond geometry (Å, °)

*D*—H⋯*A*	*D*—H	H⋯*A*	*D*⋯*A*	*D*—H⋯*A*
N2—H1*N*2⋯O1^i^	0.81 (2)	2.04 (2)	2.8318 (14)	166.6 (18)
C9—H9*A*⋯O3^ii^	0.99	2.31	3.1818 (16)	147
C9—H9*B*⋯N4^iii^	0.99	2.40	3.3462 (16)	160
C10—H10*A*⋯O2^ii^	0.95	2.56	3.4488 (16)	155

Symmetry codes: (i) 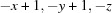; (ii) 
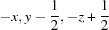
; (iii) 
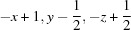
.

**Fig. 1.**
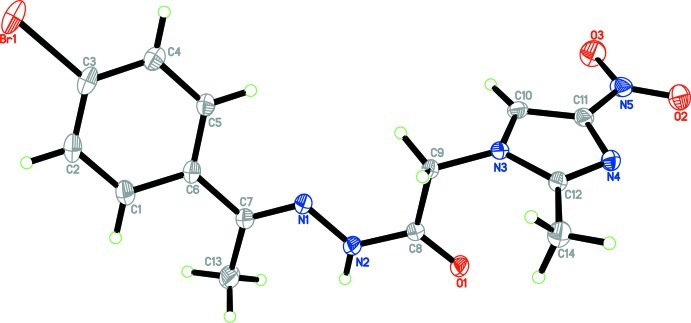
The molecular structure of the title compound showing 50% probability displacement ellipsoids for non-H atoms.

**Fig. 2.**
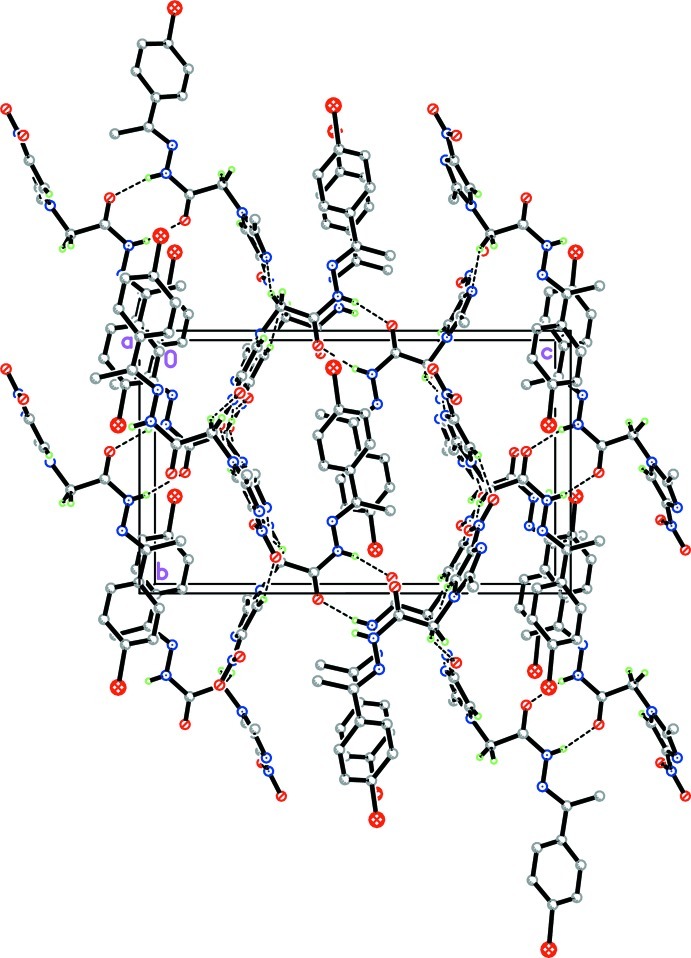
The crystal structure of the title compound, viewed along the a axis. H atoms not involved in hydrogen bonds (dashed lines) have been omitted for clarity.

## supplementary crystallographic information

### Comment

The chemistry of imidazole derivatives has been the subject of much interest due to their importance in various applications and also due to their widespread potential as well as proven biological and pharmacological activities (Priya & Kalluraya, 2005). Various applications of imidazoles have been listed in the literature with functions as widely divergent as antidepressant agents (Krapcho & Turk, 1966), as a marker for imaging tumor hypoxia( Chu *et al.*, 2004), and in antibacterial activity (Khalafi-Nezhad *et al.*, 2005). In view of the obvious importance of imidazole derivatives as potential pharmacological agents, herein we report the crystal structure of the above imidazole derivative.

In the title molecule, Fig. 1, the mean plane of the imidazole ring (N3/N4/C10-C12, r.m.s deviation = 0.004 Å) forms a dihedral angle of 58.13 (7)° with the phenyl ring (C1-C6). Bond lengths (Allen *et al.*, 1987) and angles are within normal ranges. A short Br1···Br1 contact of 3.4932 (2) Å also occurs. In the crystal (Fig. 2), molecules are linked *via* intermolecular N2–H1N2···O1, C9–H9A···O3, C9–H9B···N4 and C10–H10A···O2 hydrogen bonds (Table 1) into a three-dimensional network.

### Experimental

The title compound was synthesized by refluxing a mixture of 2-(2-methyl-4-nitro-1*H*-imidazol-1-yl)acethydrazide (0.1 mol) and 1-(4-bromophenyl)ethanone (0.1 mol) in glacial acetic acid for 1 hour. After cooling the reaction mixture to room temperature and evaporation of the solvent under reduced pressure, the solid separated was filtered, washed with water and dried. The recrystallization of the sample was done using an ethanol-dioxane (1:1 *v*/*v*) mixture. The melting point of the compound was found to be 549 K. The slow evaporation of the ethanol-dioxane mixture of the compound gave crystals suitable for X-ray analysis.

### Refinement

Atom H1N2 was located in a difference Fourier map and refined freely [N–H = 0.81 (2) Å]. All other hydrogen atoms were positioned geometrically and refined using a riding model with C–H = 0.95-0.99 Å and *U*_iso_(H) = 1.2 or 1.5 *U*_eq_(C). A rotating-group model was applied for the methyl groups.

### Crystal data

**Table:**

C_14_H_14_BrN_5_O_3_	*F*(000) = 768
*M**_r_* = 380.21	*D*_x_ = 1.610 Mg m^−^^3^
Monoclinic, *P*2_1_/*c*	Mo *K*α radiation, λ = 0.71073 Å
Hall symbol: -P 2ybc	Cell parameters from 9969 reflections
*a* = 8.4176 (1) Å	θ = 2.3–33.5°
*b* = 10.6541 (1) Å	µ = 2.64 mm^−^^1^
*c* = 17.4933 (2) Å	*T* = 100 K
β = 90.100 (1)°	Block, yellow
*V* = 1568.83 (3) Å^3^	0.37 × 0.33 × 0.15 mm
*Z* = 4	

### Data collection

**Table:**

Bruker SMART APEXII CCD area-detector diffractometer	6320 independent reflections
Radiation source: fine-focus sealed tube	5026 reflections with *I* > 2σ(*I*)
Graphite monochromator	*R*_int_ = 0.040
φ and ω scans	θ_max_ = 33.9°, θ_min_ = 2.2°
Absorption correction: multi-scan (*SADABS*; Bruker, 2009)	*h* = −13→13
*T*_min_ = 0.441, *T*_max_ = 0.701	*k* = −16→16
45099 measured reflections	*l* = −27→27

### Refinement

**Table:**

Refinement on *F*^2^	Primary atom site location: structure-invariant direct methods
Least-squares matrix: full	Secondary atom site location: difference Fourier map
*R*[*F*^2^ > 2σ(*F*^2^)] = 0.032	Hydrogen site location: inferred from neighbouring sites
*wR*(*F*^2^) = 0.082	H atoms treated by a mixture of independent and constrained refinement
*S* = 1.02	*w* = 1/[σ^2^(*F*_o_^2^) + (0.0423*P*)^2^ + 0.4612*P*] where *P* = (*F*_o_^2^ + 2*F*_c_^2^)/3
6320 reflections	(Δ/σ)_max_ = 0.001
214 parameters	Δρ_max_ = 0.88 e Å^−^^3^
0 restraints	Δρ_min_ = −0.66 e Å^−^^3^

### Special details

**Table:**

Experimental. The crystal was placed in the cold stream of an Oxford Cryosystems Cobra open-flow nitrogen cryostat (Cosier & Glazer, 1986) operating at 100.0 (1) K.
Geometry. All esds (except the esd in the dihedral angle between two l.s. planes) are estimated using the full covariance matrix. The cell esds are taken into account individually in the estimation of esds in distances, angles and torsion angles; correlations between esds in cell parameters are only used when they are defined by crystal symmetry. An approximate (isotropic) treatment of cell esds is used for estimating esds involving l.s. planes.
Refinement. Refinement of F^2^ against ALL reflections. The weighted R-factor wR and goodness of fit S are based on F^2^, conventional R-factors R are based on F, with F set to zero for negative F^2^. The threshold expression of F^2^ > 2sigma(F^2^) is used only for calculating R-factors(gt) etc. and is not relevant to the choice of reflections for refinement. R-factors based on F^2^ are statistically about twice as large as those based on F, and R- factors based on ALL data will be even larger.

### Fractional atomic coordinates and isotropic or equivalent isotropic displacement parameters (Å^2^)

**Table:**

	*x*	*y*	*z*	*U*_iso_*/*U*_eq_	
Br1	0.021322 (19)	−0.359303 (13)	0.050226 (9)	0.03144 (5)	
O1	0.45656 (12)	0.54105 (9)	0.08914 (5)	0.02360 (19)	
O2	0.13283 (13)	0.85764 (9)	0.31753 (6)	0.0269 (2)	
O3	−0.04787 (11)	0.73832 (11)	0.26628 (7)	0.0345 (3)	
N1	0.33805 (12)	0.23194 (9)	0.05430 (6)	0.01687 (18)	
N2	0.40021 (13)	0.34967 (10)	0.04122 (6)	0.01891 (19)	
N3	0.31916 (11)	0.50038 (9)	0.22432 (6)	0.01478 (17)	
N4	0.36372 (12)	0.68190 (10)	0.28408 (6)	0.01722 (18)	
N5	0.09082 (13)	0.76031 (10)	0.28541 (7)	0.0215 (2)	
C1	0.29424 (16)	−0.06979 (12)	−0.03559 (7)	0.0221 (2)	
H1A	0.3658	−0.0588	−0.0770	0.026*	
C2	0.22669 (16)	−0.18701 (12)	−0.02307 (8)	0.0241 (2)	
H2A	0.2518	−0.2559	−0.0553	0.029*	
C3	0.12217 (16)	−0.20161 (12)	0.03723 (8)	0.0229 (2)	
C4	0.08571 (16)	−0.10281 (12)	0.08621 (7)	0.0212 (2)	
H4A	0.0153	−0.1149	0.1280	0.025*	
C5	0.15433 (14)	0.01392 (11)	0.07288 (7)	0.0184 (2)	
H5A	0.1300	0.0822	0.1058	0.022*	
C6	0.25865 (14)	0.03226 (11)	0.01173 (7)	0.0181 (2)	
C7	0.32849 (15)	0.15744 (11)	−0.00367 (7)	0.0182 (2)	
C8	0.40582 (14)	0.43392 (11)	0.09867 (7)	0.0168 (2)	
C9	0.35054 (14)	0.39082 (11)	0.17701 (7)	0.0163 (2)	
H9A	0.2528	0.3399	0.1719	0.020*	
H9B	0.4335	0.3382	0.2012	0.020*	
C10	0.17458 (14)	0.55668 (11)	0.23059 (7)	0.0171 (2)	
H10A	0.0746	0.5263	0.2134	0.021*	
C11	0.20643 (14)	0.66705 (11)	0.26741 (7)	0.0172 (2)	
C12	0.43050 (13)	0.57897 (11)	0.25673 (6)	0.01558 (19)	
C13	0.38107 (18)	0.19047 (14)	−0.08367 (7)	0.0267 (3)	
H13A	0.3421	0.2744	−0.0969	0.040*	
H13B	0.3378	0.1290	−0.1198	0.040*	
H13C	0.4974	0.1894	−0.0862	0.040*	
C14	0.60238 (15)	0.54790 (13)	0.26074 (8)	0.0226 (2)	
H14A	0.6556	0.6059	0.2959	0.034*	
H14B	0.6494	0.5557	0.2098	0.034*	
H14C	0.6155	0.4616	0.2792	0.034*	
H1N2	0.436 (2)	0.3704 (17)	0.0004 (12)	0.028 (5)*	

### Atomic displacement parameters (Å^2^)

**Table:**

	*U*^11^	*U*^22^	*U*^33^	*U*^12^	*U*^13^	*U*^23^
Br1	0.03837 (9)	0.01604 (7)	0.03988 (9)	−0.00786 (5)	−0.01265 (6)	0.00296 (5)
O1	0.0347 (5)	0.0166 (4)	0.0195 (4)	−0.0089 (4)	0.0077 (4)	−0.0024 (3)
O2	0.0300 (5)	0.0167 (4)	0.0342 (5)	0.0039 (3)	0.0041 (4)	−0.0044 (4)
O3	0.0162 (4)	0.0334 (6)	0.0537 (7)	0.0041 (4)	0.0052 (4)	−0.0103 (5)
N1	0.0189 (4)	0.0138 (4)	0.0179 (4)	−0.0020 (3)	0.0007 (3)	−0.0014 (3)
N2	0.0244 (5)	0.0161 (4)	0.0162 (4)	−0.0052 (4)	0.0044 (4)	−0.0017 (4)
N3	0.0153 (4)	0.0130 (4)	0.0160 (4)	−0.0008 (3)	0.0029 (3)	−0.0008 (3)
N4	0.0176 (4)	0.0152 (4)	0.0189 (4)	0.0000 (3)	0.0009 (4)	−0.0017 (3)
N5	0.0204 (5)	0.0181 (5)	0.0261 (5)	0.0030 (4)	0.0068 (4)	0.0003 (4)
C1	0.0245 (6)	0.0194 (5)	0.0222 (5)	0.0008 (4)	−0.0012 (5)	−0.0056 (4)
C2	0.0272 (6)	0.0159 (5)	0.0291 (6)	0.0019 (5)	−0.0055 (5)	−0.0065 (5)
C3	0.0255 (6)	0.0147 (5)	0.0284 (6)	−0.0028 (4)	−0.0095 (5)	0.0003 (4)
C4	0.0241 (6)	0.0183 (5)	0.0212 (5)	−0.0036 (4)	−0.0030 (4)	0.0009 (4)
C5	0.0213 (5)	0.0156 (5)	0.0183 (5)	−0.0013 (4)	−0.0020 (4)	−0.0024 (4)
C6	0.0201 (5)	0.0149 (5)	0.0192 (5)	−0.0007 (4)	−0.0024 (4)	−0.0030 (4)
C7	0.0200 (5)	0.0168 (5)	0.0176 (5)	−0.0017 (4)	0.0007 (4)	−0.0028 (4)
C8	0.0182 (5)	0.0154 (5)	0.0168 (5)	−0.0018 (4)	0.0030 (4)	−0.0012 (4)
C9	0.0199 (5)	0.0116 (4)	0.0175 (5)	−0.0016 (4)	0.0035 (4)	−0.0016 (4)
C10	0.0145 (5)	0.0167 (5)	0.0201 (5)	−0.0011 (4)	0.0033 (4)	0.0007 (4)
C11	0.0172 (5)	0.0148 (5)	0.0197 (5)	0.0011 (4)	0.0040 (4)	−0.0004 (4)
C12	0.0165 (5)	0.0140 (4)	0.0163 (5)	−0.0002 (4)	0.0002 (4)	−0.0004 (4)
C13	0.0371 (7)	0.0252 (6)	0.0178 (5)	−0.0095 (5)	0.0034 (5)	−0.0032 (5)
C14	0.0162 (5)	0.0221 (6)	0.0296 (6)	0.0026 (4)	−0.0041 (5)	−0.0036 (5)

### Geometric parameters (Å, º)

**Table:**

Br1—C3	1.8962 (13)	C3—C4	1.3919 (19)
O1—C8	1.2302 (14)	C4—C5	1.3912 (17)
O2—N5	1.2310 (15)	C4—H4A	0.9500
O3—N5	1.2363 (15)	C5—C6	1.3989 (17)
N1—C7	1.2901 (15)	C5—H5A	0.9500
N1—N2	1.3783 (14)	C6—C7	1.4824 (17)
N2—C8	1.3483 (15)	C7—C13	1.5102 (18)
N2—H1N2	0.81 (2)	C8—C9	1.5190 (16)
N3—C10	1.3614 (15)	C9—H9A	0.9900
N3—C12	1.3779 (15)	C9—H9B	0.9900
N3—C9	1.4554 (15)	C10—C11	1.3671 (17)
N4—C12	1.3223 (15)	C10—H10A	0.9500
N4—C11	1.3644 (16)	C12—C14	1.4857 (16)
N5—C11	1.4264 (15)	C13—H13A	0.9800
C1—C2	1.3898 (19)	C13—H13B	0.9800
C1—C6	1.3992 (17)	C13—H13C	0.9800
C1—H1A	0.9500	C14—H14A	0.9800
C2—C3	1.383 (2)	C14—H14B	0.9800
C2—H2A	0.9500	C14—H14C	0.9800
			
C7—N1—N2	116.91 (10)	C6—C7—C13	119.67 (10)
C8—N2—N1	119.67 (10)	O1—C8—N2	121.89 (11)
C8—N2—H1N2	117.7 (13)	O1—C8—C9	120.65 (10)
N1—N2—H1N2	122.6 (13)	N2—C8—C9	117.44 (10)
C10—N3—C12	107.86 (9)	N3—C9—C8	109.07 (9)
C10—N3—C9	124.22 (10)	N3—C9—H9A	109.9
C12—N3—C9	126.68 (10)	C8—C9—H9A	109.9
C12—N4—C11	103.86 (10)	N3—C9—H9B	109.9
O2—N5—O3	123.61 (11)	C8—C9—H9B	109.9
O2—N5—C11	119.47 (11)	H9A—C9—H9B	108.3
O3—N5—C11	116.91 (11)	N3—C10—C11	104.02 (10)
C2—C1—C6	121.13 (12)	N3—C10—H10A	128.0
C2—C1—H1A	119.4	C11—C10—H10A	128.0
C6—C1—H1A	119.4	N4—C11—C10	112.94 (10)
C3—C2—C1	118.82 (12)	N4—C11—N5	122.29 (11)
C3—C2—H2A	120.6	C10—C11—N5	124.74 (11)
C1—C2—H2A	120.6	N4—C12—N3	111.32 (10)
C2—C3—C4	121.70 (12)	N4—C12—C14	125.61 (11)
C2—C3—Br1	118.46 (10)	N3—C12—C14	123.07 (10)
C4—C3—Br1	119.80 (11)	C7—C13—H13A	109.5
C5—C4—C3	118.74 (12)	C7—C13—H13B	109.5
C5—C4—H4A	120.6	H13A—C13—H13B	109.5
C3—C4—H4A	120.6	C7—C13—H13C	109.5
C4—C5—C6	120.96 (11)	H13A—C13—H13C	109.5
C4—C5—H5A	119.5	H13B—C13—H13C	109.5
C6—C5—H5A	119.5	C12—C14—H14A	109.5
C5—C6—C1	118.63 (11)	C12—C14—H14B	109.5
C5—C6—C7	120.95 (11)	H14A—C14—H14B	109.5
C1—C6—C7	120.41 (11)	C12—C14—H14C	109.5
N1—C7—C6	115.78 (11)	H14A—C14—H14C	109.5
N1—C7—C13	124.54 (11)	H14B—C14—H14C	109.5
			
C7—N1—N2—C8	176.66 (12)	C12—N3—C9—C8	−73.37 (14)
C6—C1—C2—C3	0.25 (19)	O1—C8—C9—N3	18.68 (16)
C1—C2—C3—C4	−1.2 (2)	N2—C8—C9—N3	−162.77 (10)
C1—C2—C3—Br1	176.42 (10)	C12—N3—C10—C11	−0.47 (13)
C2—C3—C4—C5	1.3 (2)	C9—N3—C10—C11	−168.42 (10)
Br1—C3—C4—C5	−176.37 (9)	C12—N4—C11—C10	0.27 (14)
C3—C4—C5—C6	−0.28 (19)	C12—N4—C11—N5	−178.11 (11)
C4—C5—C6—C1	−0.66 (18)	N3—C10—C11—N4	0.13 (14)
C4—C5—C6—C7	178.40 (11)	N3—C10—C11—N5	178.47 (11)
C2—C1—C6—C5	0.68 (19)	O2—N5—C11—N4	−0.47 (18)
C2—C1—C6—C7	−178.39 (12)	O3—N5—C11—N4	178.49 (12)
N2—N1—C7—C6	−178.98 (10)	O2—N5—C11—C10	−178.65 (12)
N2—N1—C7—C13	0.29 (18)	O3—N5—C11—C10	0.30 (19)
C5—C6—C7—N1	24.91 (17)	C11—N4—C12—N3	−0.58 (13)
C1—C6—C7—N1	−156.05 (12)	C11—N4—C12—C14	−179.49 (12)
C5—C6—C7—C13	−154.40 (12)	C10—N3—C12—N4	0.69 (13)
C1—C6—C7—C13	24.64 (18)	C9—N3—C12—N4	168.26 (11)
N1—N2—C8—O1	−178.13 (11)	C10—N3—C12—C14	179.63 (11)
N1—N2—C8—C9	3.34 (17)	C9—N3—C12—C14	−12.80 (18)
C10—N3—C9—C8	92.28 (13)		

### Hydrogen-bond geometry (Å, º)

**Table:**

*D*—H···*A*	*D*—H	H···*A*	*D*···*A*	*D*—H···*A*
N2—H1*N*2···O1^i^	0.81 (2)	2.04 (2)	2.8318 (14)	166.6 (18)
C9—H9*A*···O3^ii^	0.99	2.31	3.1818 (16)	147
C9—H9*B*···N4^iii^	0.99	2.40	3.3462 (16)	160
C10—H10*A*···O2^ii^	0.95	2.56	3.4488 (16)	155

Symmetry codes: (i) −*x*+1, −*y*+1, −*z*; (ii) −*x*, *y*−1/2, −*z*+1/2; (iii) −*x*+1, *y*−1/2, −*z*+1/2.
